# 1-(2-Phenyl­benz­yl)-3-(2,4,6-trimethyl­benz­yl)imidazolidinium bromide

**DOI:** 10.1107/S1600536808042086

**Published:** 2008-12-13

**Authors:** Hakan Arslan, Don VanDerveer, Sedat Yaşar, İsmail Özdemir, Bekir Çetinkaya

**Affiliations:** a Department of Natural Sciences, Fayetteville State University, Fayetteville, NC 28301, USA; b Department of Chemistry, Faculty of Pharmacy, Mersin University, Mersin, TR 33169, Turkey; c Department of Chemistry, Clemson University, Clemson, SC 29634, USA; d Department of Chemistry, Faculty of Science and Arts, İnönü University, Malatya, TR 44280, Turkey; e Department of Chemistry, Faculty of Science, Ege University, Bornova-İzmir, TR 35100, Turkey

## Abstract

In the title salt, C_26_H_29_N_2_^+^·Br^−^, which may serve as a precursor for *N*-heterocyclic carbenes, the imidazolidine ring adopts a twist conformation with a pseudo-twofold axis passing through the N—C—N carbon and the opposite C—C bond. The N—C—N bond angle [113.0 (4)°] and C—N bond lengths [1.313 (6) and 1.305 (6) Å] confirm the existence of strong resonance in this part of the mol­ecule. In the crystal, a C—H⋯Br inter­action is present. The dihedral angle between the biphenyl rings is 64.3 (2)° and the phenyl rings make angles of 76.6 (3) and 18.5 (3)° with the plane of the imidazolidine ring.

## Related literature

For the synthesis, see: Özdemir *et al.* (2005*b* ▶,*d* ▶). For general background, see: Herrmann (2002 ▶); Scott & Nolan (2005 ▶). For puckering and asymmetry parameters, see: Cremer & Pople (1975 ▶); Nardelli (1983 ▶). For related compounds, see: Arduengo *et al.* (1995*a* ▶,*b* ▶); Hagos *et al.* (2008 ▶). For bond-length data, see: Allen *et al.* (1987 ▶). For related literature, see: Arslan *et al.* (2004*a* ▶,*b* ▶, 2005*a* ▶,*b* ▶, 2007*a* ▶,*b* ▶,*c* ▶); Cavell & Guinness (2004 ▶); Hahn (2006 ▶); Kirmse (2004 ▶); Nair *et al.* (2004 ▶); Rangits & Kollar (2006 ▶); Richmond (2000 ▶); Özdemir *et al.* (2004 ▶, 2005*a* ▶,*c* ▶).
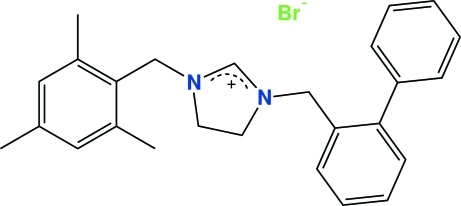


## Experimental

### 

#### Crystal data

C_26_H_29_N_2_^+^·Br^−^
*M* *_r_* = 449.42
Monoclinic, 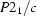

*a* = 18.626 (4) Å
*b* = 13.793 (3) Å
*c* = 8.8181 (18) Å
β = 95.08 (3)°
*V* = 2256.6 (8) Å^3^
*Z* = 4
Mo *K*α radiation
μ = 1.84 mm^−1^
*T* = 183 (2) K
0.36 × 0.19 × 0.12 mm

#### Data collection

Rigaku AFC-8S Mercury CCD diffractometer
Absorption correction: multi-scan (*REQAB*; Jacobson, 1998 ▶) *T*_min_ = 0.615, *T*_max_ = 0.798
18802 measured reflections
4010 independent reflections
3051 reflections with *I* > 2σ(*I*)
*R*_int_ = 0.060

#### Refinement

*R*[*F* ^2^ > 2σ(*F* ^2^)] = 0.078
*wR*(*F* ^2^) = 0.241
*S* = 1.09
4010 reflections
266 parameters
H-atom parameters constrained
Δρ_max_ = 0.75 e Å^−3^
Δρ_min_ = −1.13 e Å^−3^

### 

Data collection: *CrystalClear* (Rigaku/MSC, 2006 ▶); cell refinement: *CrystalClear*; data reduction: *CrystalClear* (Rigaku/MSC, 2006 ▶); program(s) used to solve structure: *SHELXTL* (Sheldrick, 2008 ▶); program(s) used to refine structure: *SHELXTL*; molecular graphics: *SHELXTL*; software used to prepare material for publication: *SHELXTL*.

## Supplementary Material

Crystal structure: contains datablocks global, I. DOI: 10.1107/S1600536808042086/hg2448sup1.cif

Structure factors: contains datablocks I. DOI: 10.1107/S1600536808042086/hg2448Isup2.hkl

Additional supplementary materials:  crystallographic information; 3D view; checkCIF report

## Figures and Tables

**Table 1:** Hydrogen-bond geometry (Å, °)

*D*—H⋯*A*	*D*—H	H⋯*A*	*D*⋯*A*	*D*—H⋯*A*
C3—H3⋯Br1^i^	0.96	2.52	3.472 (5)	170

Symmetry code: (i) 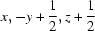.

**Fig. 1.**
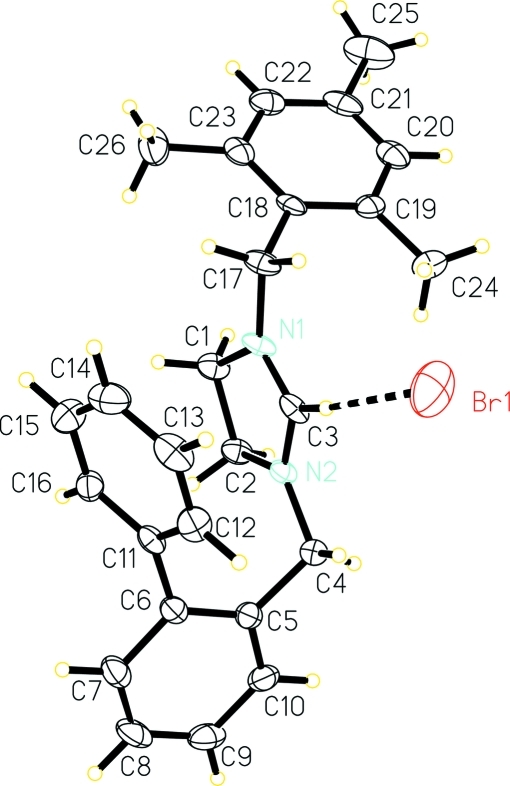
The molecular structure of the title compound, showing the atom-numbering scheme and displacement ellipsoids drawn at the 50% probability level.

**Fig. 2.**
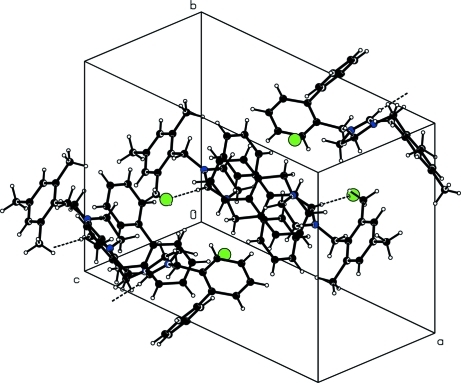
A view of the packing diagram of (I). Hydrogen bonds are shown as dashed lines.

**Fig. 3.**
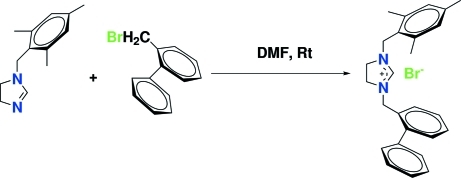
Reaction scheme.

## supplementary crystallographic information

### Comment

Recently, *N*-heterocyclic carbene compounds have become universal ligands in inorganic and organometallic chemistry (Hahn, 2006; Kirmse, 2004). The role of *N*-heterocyclic carbenes as alternative compounds to tertiary phosphines in homogeneous catalysis is now well established (Scott & Nolan, 2005; Herrmann, 2002). At the same time, it has become increasingly apparent that carbenes (and/or their imidazolium precursors) are quite susceptible to metal induced bond activation reactions, which may be crucial to both catalytic activity and possible deactivation pathways. The chemistry of *N*-heterocyclic carbenes has become a major area of research as these stable carbenes (Nair *et al.*, 2004) have proven to be outstanding ligands for transition metals (Cavell & Guinness, 2004), as well as potent nucleophilic organic catalysts (Rangits & Kollar, 2006). In this field, the conjugate acids play a very important role. Indeed, they are by far the most frequently used precursors of *N*-heterocyclic carbenes, and oxidative addition of the CH bond to transition metal centers can even directly generate the carbine complexes (Herrmann, 2002). Moreover, the conjugate acids of *N*-heterocyclic carbenes (H^+^) have found numerous applications as ionic liquids (Richmond, 2000), which are important components of green chemistry.

We have previously reported the use of *in situ* formed imidazolidin-2-ylidene, tetrahydropyrimidin-2-ylidene and tetrahydrodiazepin-2-ylidene palladium (II) systems that exhibit high activity for various coupling reactions of aryl bromides and aryl chlorides (Özdemir *et al.*, 2005*a*, 2005*b*). In order to obtain more stable, efficient and active systems, we have also investigated benzo-annelated derivatives (Özdemir *et al.*, 2004). Recently our group reported that novel complexes of rhodium(I) 1,3-dialkyimidazolidin-2-ylidenes gave secondary alcohols in good yields by the addition of phenylboronic acid to aldehydes (Özdemir *et al.*, 2005*c*). In recent years, we also investigated the crystal structures of new *N*-heterocyclic carbene derivatives (Arslan *et al.*, 2004*a*, 2004*b*, 2005*a*, 2005*b*, 2007*a*, 2007*b*, 2007*c*).

Due to our interest in preparing stable ligands for selected catalytic processes, we prepared 1-(2,4,6-trimethylbenzyl)-3-(2-phenylbenzyl)-imidazolidinium bromide, (I) as a carbene precursor. The title compound was purified by re-crystallization from an ethanol:diethylether mixture (2:1) and characterized by elemental analysis, ^1^H and ^13^C-NMR and IR spectroscopy (Özdemir *et al.* 2005d). The analytical and spectroscopic data are consistent with the proposed structure given in Scheme1. The structure consists of a 1-(2,4,6-trimethylbenzyl)-3-(2-phenylbenzyl)-imidazolidinium cation and a Br^-^anion. The structure of (I) is shown in Fig. 1.

The crystallographic data for (I) will provide valuable information for assessing its electronic conjugation properties and a possible intra- and inter-molecular hydrogen bond, which may be correlated with the reactivity of the carben carbonatom. N—C_carben_—N bond angles and C_carben_—N bond lengths are related with the proximity of the proton in the carben compounds (Arduengo *et al.*, 1995*b*). Though N—C_carben_—N bond angles are observed as 101.4 (2) ° and 108.7 (4) ° for 1,3-dimesityl-2,3-dihydro-1*H*-imidazole-2-ylidene and 1,3-dimesityl-1*H*-imidazol-3-ium, respectively, (Arduengo *et al.*, 1995*b*), N—C_carben_—N bond angle is 113.0 (4) ° in the title compound. According to this result, the larger N—C_carben_—N bond angle is more relaxed than the other carben derivatives. In addition, C3—H3···Br1 intermolecular hydrogen bond and C_carben_—N bond lengths have confirmed this result (Arduengo *et al.*, 1995*a*, 1995*b*). The same geometric behaviors are observed for 1,3-dimesitylimidazolidinium tetrachloridogold(III) and 1,3-dimesityl-4,5-dihydro-1*H*-imidazol-3-ium chloride where the N—C_carben_—N bond angles are 113.9 (6) ° and 113.1 (4) °, respectively (Hagos *et al.*, 2008; Arduengo *et al.*, 1995*b*).

The C_carben_—N bond distances in the imidazolidine ring are not the expected single and double bond lengths as reported in other imidazolidine derivatives (Arduengo *et al.*, 1995*a*, 1995*b*). Although the C_carben_—N bond distances (N1—C3 = 1.313 (6) Å and N2—C3 = 1.305 (6) Å) in the title compound are shorter than the average C_carben_—N bond length for free carbene derivatives which have imidazolidine rings is 1.360 Å. The C—N bond lengths have intermediate values between the single and double C—N bond lengths (Allen *et al.*, 1987) and agree with C_carben_—N bond lengths values of 1,3-dimesitylimidazolidinium tetrachloridogold(III) and 1,3-dimesityl-4,5-dihydro-1*H*-imidazol-3-ium chloride (1.294 (7) and 1.320 (8) Å) (Hagos *et al.*, 2008), (1.327 (5) and 1.310 (5) Å) (Arduengo *et al.*, 1995*b*), respectively. Also, the sum of the bond angles around N1 (360.0 °) and N2 (360.0 °) indicate *sp*^2 ^hybridization. These results can be explained by existence of resonance in this part of the molecule. All the other bond lengths are in normal ranges (Allen *et al.*, 1987).

The deviations from planarity of imidazolidine ring are C1 0.056 (5), C2 0.051 (5), N2 0.027 (4), C3 0.008 (5) and N1 0.039 (4) Å. The puckering parameters (Cremer & Pople, 1975) and the smallest displacement asymmetry parameters (Nardelli, 1983) for the imidazolidine ringare *q*_2_ = 0.090 (5) Å, *φ*_2_= 46 (3)° and Δ*C*_2_(C3) = 13.0 (5), Δ*C*_s_(C3) = 5.1 (5). According to these results, the imidazolidine ring adopts a twisted conformation with a pseudo-twofold axis passing through C3 and the C1—C2 bond.

The crystal packing is shown in Fig. 2. The crystal packing is dominated by intermolecular C3—H3···Br1 (*x*,1/2 - *y*,1/2 + *z*) hydrogen bonds, with H···Br = 2.52 Å and C—H···Br 170 ^o ^(Table 1).

### Experimental

To a solution of 1-(2,4,6-trimethylbenzyl) imidazoline (2.02 g, 10 mmol) in DMF (5 ml) was added slowly 2-phenylbenzyl bromide (2.49 g, 10.07 mmol) at 25 °C and the resulting mixture was stirred at room temperature for 8 h (Fig. 3). Diethylether (15 ml) was added to obtain a white crystalline solid which was filtered off. The solid was washed with diethylether (3 *x* 10 ml), dried under vacuum and the crude product was recrystallized from ethanol:diethylether mixture (2:1) (Özdemir *et al., *2005 d). *M*.p.: 241–241.5 °C. Yield: 4.22 g, 94%. *υ*(CN) = 1444 cm^-1. 1^H NMR (*δ*, CDCl_3_): 2.26 and 2.27 (s, 9H, CH_2_C_6_H_2_(C*H*_3_)_3_-2,4,6), 6.32 (s, 2H, CH_2_C_6_*H*_2_(CH_3_)_3_-2,4,6), 4.66 (s, 2H, C*H*_2_C_6_H_2_(CH_3_)_3_-2,4,6), 3.51 and 3.61 (m, 4H, NC*H*_2_C*H*_2_N), 4.88 (s, 2H, C*H*_2_C_6_H_4_(*o*-C_6_H_5_), 7.21–7.54 (m, 9H,CH_2_C_6_*H*_4_(*o*-C_6_*H*_5_), 8.78 (s, 1H, NC*H*N). ^13^C{H}NMR (*δ*, CDCl_3_): 20.5 and 21.2 (CH_2_C_6_H_2_(*C*H_3_)_3_-2,4,6), 125.5, 128.9, 138.0, and 139.3 (CH_2_*C*_6_H_2_(CH_3_)_3_-2,4,6), 46.5 (*C*H_2_C_6_H_2_(CH_3_)_3_-2,4,6), 47.9 and 48.3 (N*C*H_2_*C*H_2_N), 50.5 (*C*H_2_C_6_H_4_(*o*-C_6_H_5_), 127.9, 128.7, 129.0, 129.4, 129.9, 130.0, 130.4, 130.8, 139.9, 142.5 (CH_2_*C*_6_H_4_(*o*-*C*_6_H_5_), 157.5 (N*C*HN). Found: C 69.45, H 6.51, N 6.26%. Calcd. for C_26_H_29_N_2_Br: C 69.48, H 6.50, N 6.23%.

### Crystal data

**Table:**

C_26_H_29_N_2_^+^·Br^−^	*F*(000) = 936
*M**_r_* = 449.42	*D*_x_ = 1.323 Mg m^−^^3^
Monoclinic, *P*2_1_/*c*	Mo *K*α radiation, λ = 0.71073 Å
Hall symbol: -P 2ybc	Cell parameters from 8750 reflections
*a* = 18.626 (4) Å	θ = 2.2–26.0°
*b* = 13.793 (3) Å	µ = 1.84 mm^−^^1^
*c* = 8.8181 (18) Å	*T* = 183 K
β = 95.08 (3)°	Rod, colorless
*V* = 2256.6 (8) Å^3^	0.36 × 0.19 × 0.12 mm
*Z* = 4	

### Data collection

**Table:**

Rigaku AFC-8S Mercury CCD diffractometer	4010 independent reflections
Radiation source: Sealed Tube	3051 reflections with *I* > 2σ(*I*)
Graphite Monochromator	*R*_int_ = 0.060
Detector resolution: 14.6199 pixels mm^-1^	θ_max_ = 25.1°, θ_min_ = 2.2°
ω scans	*h* = −22→22
Absorption correction: multi-scan (REQAB; Jacobson, 1998)	*k* = −16→16
*T*_min_ = 0.615, *T*_max_ = 0.798	*l* = −10→10
18802 measured reflections	

### Refinement

**Table:**

Refinement on *F*^2^	Primary atom site location: structure-invariant direct methods
Least-squares matrix: full	Secondary atom site location: difference Fourier map
*R*[*F*^2^ > 2σ(*F*^2^)] = 0.078	Hydrogen site location: inferred from neighbouring sites
*wR*(*F*^2^) = 0.241	H-atom parameters constrained
*S* = 1.09	*w* = 1/[σ^2^(*F*_o_^2^) + (0.1604*P*)^2^ + 1.5464*P*] where *P* = (*F*_o_^2^ + 2*F*_c_^2^)/3
4010 reflections	(Δ/σ)_max_ = 0.001
266 parameters	Δρ_max_ = 0.75 e Å^−^^3^
0 restraints	Δρ_min_ = −1.13 e Å^−^^3^

### Special details

**Table:**

Geometry. All e.s.d.'s (except the e.s.d. in the dihedral angle between two l.s. planes) are estimated using the full covariance matrix. The cell e.s.d.'s are taken into account individually in the estimation of e.s.d.'s in distances, angles and torsion angles; correlations between e.s.d.'s in cell parameters are only used when they are defined by crystal symmetry. An approximate (isotropic) treatment of cell e.s.d.'s is used for estimating e.s.d.'s involving l.s. planes.
Refinement. Refinement of *F*^2^ against ALL reflections. The weighted *R*-factor *wR* and goodness of fit *S* are based on *F*^2^, conventional *R*-factors *R* are based on *F*, with *F* set to zero for negative *F*^2^. The threshold expression of *F*^2^ > σ(*F*^2^) is used only for calculating *R*-factors(gt) *etc*. and is not relevant to the choice of reflections for refinement. *R*-factors based on *F*^2^ are statistically about twice as large as those based on *F*, and *R*- factors based on ALL data will be even larger.

### Fractional atomic coordinates and isotropic or equivalent isotropic displacement parameters (Å^2^)

**Table:**

	*x*	*y*	*z*	*U*_iso_*/*U*_eq_	
Br1	0.26262 (5)	0.44009 (6)	0.82641 (7)	0.0611 (3)	
N1	0.18896 (19)	0.0693 (3)	0.8527 (4)	0.0237 (8)	
N2	0.28839 (18)	0.1536 (3)	0.8601 (4)	0.0199 (8)	
C1	0.1919 (2)	0.0981 (4)	0.6940 (5)	0.0246 (10)	
H1A	0.1486	0.1310	0.6561	0.030*	
H1B	0.1990	0.0429	0.6306	0.030*	
C2	0.2573 (2)	0.1665 (3)	0.7014 (5)	0.0233 (9)	
H2A	0.2908	0.1477	0.6302	0.028*	
H2B	0.2427	0.2325	0.6820	0.028*	
C3	0.2464 (2)	0.0999 (4)	0.9361 (5)	0.0228 (9)	
H3	0.2565	0.0844	1.0420	0.027*	
C4	0.3567 (2)	0.1972 (3)	0.9213 (5)	0.0238 (9)	
H4A	0.3690	0.1732	1.0225	0.029*	
H4B	0.3508	0.2662	0.9277	0.029*	
C5	0.4179 (2)	0.1750 (3)	0.8235 (5)	0.0227 (9)	
C6	0.4328 (2)	0.0805 (3)	0.7758 (5)	0.0219 (9)	
C7	0.4901 (3)	0.0680 (4)	0.6813 (6)	0.0299 (11)	
H7	0.5011	0.0042	0.6464	0.036*	
C8	0.5299 (3)	0.1449 (5)	0.6396 (6)	0.0355 (12)	
H8	0.5685	0.1346	0.5761	0.043*	
C9	0.5154 (3)	0.2370 (4)	0.6871 (6)	0.0343 (12)	
H9	0.5437	0.2907	0.6574	0.041*	
C10	0.4585 (2)	0.2522 (4)	0.7797 (6)	0.0277 (10)	
H10	0.4480	0.3166	0.8125	0.033*	
C11	0.3903 (2)	−0.0055 (3)	0.8174 (5)	0.0214 (9)	
C12	0.3899 (3)	−0.0353 (4)	0.9702 (6)	0.0289 (11)	
H12	0.4185	−0.0014	1.0490	0.035*	
C13	0.3481 (3)	−0.1133 (4)	1.0064 (6)	0.0340 (11)	
H13	0.3475	−0.1326	1.1108	0.041*	
C14	0.3074 (3)	−0.1638 (4)	0.8945 (7)	0.0368 (12)	
H14	0.2792	−0.2185	0.9211	0.044*	
C15	0.3072 (3)	−0.1356 (4)	0.7439 (6)	0.0345 (11)	
H15	0.2786	−0.1704	0.6660	0.041*	
C16	0.3487 (2)	−0.0567 (3)	0.7052 (5)	0.0247 (10)	
H16	0.3485	−0.0375	0.6005	0.030*	
C17	0.1316 (2)	0.0098 (4)	0.9081 (6)	0.0260 (10)	
H17A	0.1407	0.0013	1.0162	0.031*	
H17B	0.1319	−0.0530	0.8614	0.031*	
C18	0.0574 (2)	0.0560 (3)	0.8730 (5)	0.0220 (9)	
C19	0.0392 (2)	0.1386 (3)	0.9524 (6)	0.0254 (10)	
C20	−0.0292 (3)	0.1791 (4)	0.9211 (6)	0.0308 (11)	
H20	−0.0419	0.2359	0.9756	0.037*	
C21	−0.0789 (2)	0.1391 (4)	0.8132 (6)	0.0326 (11)	
C22	−0.0600 (3)	0.0574 (4)	0.7337 (6)	0.0303 (11)	
H22	−0.0941	0.0300	0.6574	0.036*	
C23	0.0079 (2)	0.0142 (4)	0.7626 (5)	0.0256 (10)	
C24	0.0914 (3)	0.1861 (4)	1.0706 (7)	0.0422 (13)	
H24A	0.1256	0.2240	1.0207	0.063*	
H24B	0.1164	0.1371	1.1320	0.063*	
H24C	0.0655	0.2275	1.1341	0.063*	
C25	−0.1526 (3)	0.1846 (5)	0.7800 (9)	0.0527 (17)	
H25A	−0.1505	0.2329	0.7022	0.079*	
H25B	−0.1671	0.2143	0.8709	0.079*	
H25C	−0.1868	0.1355	0.7460	0.079*	
C26	0.0244 (3)	−0.0751 (4)	0.6756 (7)	0.0387 (12)	
H26A	−0.0163	−0.0912	0.6056	0.058*	
H26B	0.0344	−0.1279	0.7452	0.058*	
H26C	0.0656	−0.0635	0.6201	0.058*	

### Atomic displacement parameters (Å^2^)

**Table:**

	*U*^11^	*U*^22^	*U*^33^	*U*^12^	*U*^13^	*U*^23^
Br1	0.0930 (6)	0.0614 (5)	0.0280 (4)	0.0157 (4)	0.0006 (3)	−0.0021 (3)
N1	0.0173 (17)	0.032 (2)	0.023 (2)	−0.0009 (15)	0.0085 (15)	0.0097 (16)
N2	0.0164 (16)	0.028 (2)	0.0162 (17)	−0.0001 (14)	0.0043 (14)	0.0024 (15)
C1	0.023 (2)	0.030 (3)	0.020 (2)	−0.0016 (18)	0.0031 (17)	0.0082 (19)
C2	0.021 (2)	0.028 (2)	0.020 (2)	−0.0010 (18)	0.0039 (17)	0.0074 (18)
C3	0.019 (2)	0.030 (2)	0.020 (2)	0.0085 (17)	0.0062 (17)	0.0045 (18)
C4	0.020 (2)	0.027 (2)	0.025 (2)	−0.0013 (18)	0.0031 (17)	−0.0032 (19)
C5	0.020 (2)	0.032 (2)	0.016 (2)	−0.0009 (17)	0.0003 (16)	−0.0022 (18)
C6	0.0178 (19)	0.031 (2)	0.016 (2)	0.0032 (18)	−0.0004 (16)	−0.0007 (18)
C7	0.025 (2)	0.043 (3)	0.022 (2)	0.006 (2)	0.0042 (19)	−0.004 (2)
C8	0.023 (2)	0.061 (4)	0.025 (2)	0.000 (2)	0.0106 (19)	0.000 (2)
C9	0.027 (2)	0.048 (3)	0.028 (3)	−0.015 (2)	0.004 (2)	0.004 (2)
C10	0.024 (2)	0.030 (3)	0.029 (2)	−0.0073 (19)	−0.0001 (18)	−0.002 (2)
C11	0.0178 (19)	0.023 (2)	0.024 (2)	0.0070 (16)	0.0044 (16)	0.0013 (18)
C12	0.028 (2)	0.034 (3)	0.024 (2)	0.0054 (19)	−0.0028 (19)	0.002 (2)
C13	0.033 (2)	0.038 (3)	0.033 (3)	0.009 (2)	0.009 (2)	0.010 (2)
C14	0.028 (2)	0.034 (3)	0.050 (3)	−0.002 (2)	0.008 (2)	0.007 (2)
C15	0.032 (2)	0.035 (3)	0.035 (3)	−0.004 (2)	−0.001 (2)	−0.006 (2)
C16	0.026 (2)	0.025 (2)	0.023 (2)	0.0010 (17)	0.0027 (18)	−0.0029 (18)
C17	0.019 (2)	0.027 (2)	0.033 (3)	0.0019 (18)	0.0091 (19)	0.013 (2)
C18	0.017 (2)	0.022 (2)	0.029 (2)	−0.0015 (16)	0.0125 (18)	0.0053 (17)
C19	0.024 (2)	0.020 (2)	0.033 (2)	−0.0066 (17)	0.0092 (19)	0.0006 (19)
C20	0.032 (2)	0.021 (2)	0.042 (3)	0.0008 (18)	0.018 (2)	0.008 (2)
C21	0.024 (2)	0.032 (3)	0.044 (3)	0.002 (2)	0.015 (2)	0.018 (2)
C22	0.026 (2)	0.041 (3)	0.024 (2)	−0.006 (2)	0.0041 (19)	0.008 (2)
C23	0.030 (2)	0.026 (2)	0.022 (2)	−0.0017 (18)	0.0114 (18)	0.0072 (19)
C24	0.034 (3)	0.040 (3)	0.053 (4)	−0.016 (2)	0.010 (2)	−0.014 (3)
C25	0.026 (3)	0.052 (4)	0.081 (5)	0.015 (3)	0.007 (3)	0.026 (3)
C26	0.047 (3)	0.040 (3)	0.030 (3)	0.002 (2)	0.010 (2)	−0.005 (2)

### Geometric parameters (Å, °)

**Table:**

N1—C3	1.313 (6)	C13—C14	1.379 (8)
N1—C1	1.460 (6)	C13—H13	0.9600
N1—C17	1.465 (6)	C14—C15	1.384 (8)
N2—C3	1.305 (6)	C14—H14	0.9600
N2—C4	1.467 (6)	C15—C16	1.394 (7)
N2—C2	1.477 (6)	C15—H15	0.9600
C1—C2	1.538 (6)	C16—H16	0.9600
C1—H1A	0.9600	C17—C18	1.528 (6)
C1—H1B	0.9600	C17—H17A	0.9600
C2—H2A	0.9600	C17—H17B	0.9600
C2—H2B	0.9600	C18—C19	1.395 (7)
C3—H3	0.9600	C18—C23	1.404 (7)
C4—C5	1.520 (6)	C19—C20	1.395 (7)
C4—H4A	0.9600	C19—C24	1.511 (7)
C4—H4B	0.9600	C20—C21	1.383 (8)
C5—C10	1.382 (7)	C20—H20	0.9600
C5—C6	1.404 (7)	C21—C22	1.390 (8)
C6—C7	1.422 (7)	C21—C25	1.513 (7)
C6—C11	1.490 (6)	C22—C23	1.400 (7)
C7—C8	1.363 (8)	C22—H22	0.9600
C7—H7	0.9600	C23—C26	1.497 (7)
C8—C9	1.371 (8)	C24—H24A	0.9599
C8—H8	0.9600	C24—H24B	0.9599
C9—C10	1.408 (7)	C24—H24C	0.9599
C9—H9	0.9600	C25—H25A	0.9599
C10—H10	0.9600	C25—H25B	0.9599
C11—C16	1.395 (6)	C25—H25C	0.9599
C11—C12	1.409 (7)	C26—H26A	0.9599
C12—C13	1.381 (8)	C26—H26B	0.9599
C12—H12	0.9600	C26—H26C	0.9599
			
C3—N1—C1	110.6 (4)	C12—C13—H13	119.6
C3—N1—C17	125.2 (4)	C13—C14—C15	119.9 (5)
C1—N1—C17	124.2 (4)	C13—C14—H14	120.0
C3—N2—C4	125.7 (4)	C15—C14—H14	120.0
C3—N2—C2	110.6 (4)	C14—C15—C16	120.0 (5)
C4—N2—C2	123.7 (3)	C14—C15—H15	120.0
N1—C1—C2	102.9 (4)	C16—C15—H15	120.0
N1—C1—H1A	111.2	C15—C16—C11	120.5 (4)
C2—C1—H1A	111.2	C15—C16—H16	119.8
N1—C1—H1B	111.2	C11—C16—H16	119.8
C2—C1—H1B	111.2	N1—C17—C18	111.9 (4)
H1A—C1—H1B	109.1	N1—C17—H17A	109.2
N2—C2—C1	102.1 (3)	C18—C17—H17A	109.2
N2—C2—H2A	111.4	N1—C17—H17B	109.2
C1—C2—H2A	111.4	C18—C17—H17B	109.2
N2—C2—H2B	111.4	H17A—C17—H17B	107.9
C1—C2—H2B	111.4	C19—C18—C23	120.6 (4)
H2A—C2—H2B	109.2	C19—C18—C17	119.6 (4)
N2—C3—N1	113.0 (4)	C23—C18—C17	119.8 (4)
N2—C3—H3	123.5	C18—C19—C20	119.1 (4)
N1—C3—H3	123.5	C18—C19—C24	122.0 (4)
N2—C4—C5	112.2 (4)	C20—C19—C24	118.9 (5)
N2—C4—H4A	109.2	C21—C20—C19	121.4 (5)
C5—C4—H4A	109.2	C21—C20—H20	119.3
N2—C4—H4B	109.2	C19—C20—H20	119.3
C5—C4—H4B	109.2	C20—C21—C22	119.0 (4)
H4A—C4—H4B	107.9	C20—C21—C25	120.6 (5)
C10—C5—C6	120.2 (4)	C22—C21—C25	120.4 (5)
C10—C5—C4	117.4 (4)	C21—C22—C23	121.2 (5)
C6—C5—C4	122.4 (4)	C21—C22—H22	119.4
C5—C6—C7	117.9 (4)	C23—C22—H22	119.4
C5—C6—C11	122.8 (4)	C22—C23—C18	118.6 (5)
C7—C6—C11	119.3 (4)	C22—C23—C26	118.6 (5)
C8—C7—C6	121.1 (5)	C18—C23—C26	122.7 (4)
C8—C7—H7	119.4	C19—C24—H24A	109.5
C6—C7—H7	119.4	C19—C24—H24B	109.5
C7—C8—C9	120.8 (4)	H24A—C24—H24B	109.5
C7—C8—H8	119.6	C19—C24—H24C	109.5
C9—C8—H8	119.6	H24A—C24—H24C	109.5
C8—C9—C10	119.6 (5)	H24B—C24—H24C	109.5
C8—C9—H9	120.2	C21—C25—H25A	109.5
C10—C9—H9	120.2	C21—C25—H25B	109.5
C5—C10—C9	120.4 (5)	H25A—C25—H25B	109.5
C5—C10—H10	119.8	C21—C25—H25C	109.5
C9—C10—H10	119.8	H25A—C25—H25C	109.5
C16—C11—C12	118.7 (4)	H25B—C25—H25C	109.5
C16—C11—C6	120.2 (4)	C23—C26—H26A	109.5
C12—C11—C6	121.1 (4)	C23—C26—H26B	109.5
C13—C12—C11	120.0 (5)	H26A—C26—H26B	109.5
C13—C12—H12	120.0	C23—C26—H26C	109.5
C11—C12—H12	120.0	H26A—C26—H26C	109.5
C14—C13—C12	120.9 (5)	H26B—C26—H26C	109.5
C14—C13—H13	119.6		
			
C3—N1—C1—C2	−8.6 (5)	C16—C11—C12—C13	−0.6 (7)
C17—N1—C1—C2	174.6 (4)	C6—C11—C12—C13	177.8 (4)
C3—N2—C2—C1	−6.8 (5)	C11—C12—C13—C14	1.0 (7)
C4—N2—C2—C1	174.1 (4)	C12—C13—C14—C15	−0.9 (8)
N1—C1—C2—N2	8.7 (4)	C13—C14—C15—C16	0.6 (8)
C4—N2—C3—N1	−179.2 (4)	C14—C15—C16—C11	−0.2 (8)
C2—N2—C3—N1	1.7 (5)	C12—C11—C16—C15	0.2 (7)
C1—N1—C3—N2	4.7 (5)	C6—C11—C16—C15	−178.2 (4)
C17—N1—C3—N2	−178.5 (4)	C3—N1—C17—C18	125.6 (5)
C3—N2—C4—C5	128.7 (5)	C1—N1—C17—C18	−58.0 (6)
C2—N2—C4—C5	−52.3 (6)	N1—C17—C18—C19	−71.4 (6)
N2—C4—C5—C10	129.1 (4)	N1—C17—C18—C23	109.4 (5)
N2—C4—C5—C6	−49.8 (6)	C23—C18—C19—C20	0.3 (7)
C10—C5—C6—C7	−0.4 (6)	C17—C18—C19—C20	−179.0 (4)
C4—C5—C6—C7	178.5 (4)	C23—C18—C19—C24	−179.2 (5)
C10—C5—C6—C11	−179.1 (4)	C17—C18—C19—C24	1.6 (7)
C4—C5—C6—C11	−0.3 (6)	C18—C19—C20—C21	−0.1 (7)
C5—C6—C7—C8	0.6 (7)	C24—C19—C20—C21	179.4 (5)
C11—C6—C7—C8	179.4 (4)	C19—C20—C21—C22	−0.6 (7)
C6—C7—C8—C9	−0.3 (8)	C19—C20—C21—C25	−179.5 (5)
C7—C8—C9—C10	−0.2 (8)	C20—C21—C22—C23	1.0 (7)
C6—C5—C10—C9	−0.1 (7)	C25—C21—C22—C23	180.0 (5)
C4—C5—C10—C9	−179.0 (4)	C21—C22—C23—C18	−0.8 (7)
C8—C9—C10—C5	0.5 (7)	C21—C22—C23—C26	178.6 (5)
C5—C6—C11—C16	114.2 (5)	C19—C18—C23—C22	0.2 (6)
C7—C6—C11—C16	−64.5 (6)	C17—C18—C23—C22	179.4 (4)
C5—C6—C11—C12	−64.2 (6)	C19—C18—C23—C26	−179.2 (4)
C7—C6—C11—C12	117.1 (5)	C17—C18—C23—C26	0.0 (7)

### Hydrogen-bond geometry (Å, °)

**Table:**

*D*—H···*A*	*D*—H	H···*A*	*D*···*A*	*D*—H···*A*
C3—H3···Br1^i^	0.96	2.52	3.472 (5)	170

Symmetry codes: (i) *x*, −*y*+1/2, *z*+1/2.
